# Health Tract, No. 49

**Published:** 1865

**Authors:** 


### HEALTH TRACT, No. 49.

### HYDROPHOBIA.

EMINENT Europeans have said that as many persons die of hydrophobia in winter as in summer. Persons frequently become hydrophobic after having been bitten by dogs and other animals, supposed to have been perfectly sound and well. In many cases the animal is killed instantly on having bitten a person, which person sometimes became hydrophobic, and sometimes not; sometimes within a month, at others not until two years have passed away. See JOURNAL OF HEALTH for July, 1857, where a man had been bitten by a dog nine months before, and had forgotten all about it, until reminded of it accidentally on Tuesday, May 26: on Friday following he died in horrible agonies from hydrophobia. From these statements important practical inferences should be drawn: First, never allude to hydrophobia in the presence of one who has been bitten by a dog; second, if bitten by any dog, at any season of the year, under any circumstances, whether the skin be penetrated or imperceptibly grazed by the animal's tooth, let the part be cupped, or sucked an hour by one or a succession of persons, who are most perfectly certain that there is not the slightest sore or abrasion any where about the lips or tongue. Meanwhile, administer an enema, wash the parts freely in spirits of hartshorn every half-hour for three hours, and then every hour for the remainder of the day, putting fresh hartshorn in a clean saucer on each occasion; if any thing at all is eaten, let it be of the lightest, simplest kind. The object of the hartshorn, which is the strongest alkali, is to neutralize the poison, which, like almost, if not all, bites, is acid. One of the objects in eating but little, is not to use the strength of the system in digesting the food, but rather let it be employed in repelling diseased influences. In the mean time, send for a physician. Rabid saliva has no effect whatever on *unbroken* skin. Mad dogs have perfectly lucid intervals. The great John Hunter knew twenty-one persons bitten, and only one became hydrophobic. Wasp and bee stings are generally innocuous, but occasionally are fatal in a few days, showing that some persons are insusceptible of the poison. Common-sense dictates an instant determination whether the dog is mad; if so, one or more of the following symptoms are exhibited in a very exaggerated degree: fidgety; sullen; importunately affectionate; hallucinated; ardently thirsty; scratches his ear violently; paws the corners of his mouth, *without* its being permanently open; misconducts himself, and partially recovering, licks the corners; refuses his natural food; appetite is depraved; is in sensible to pain; voice always strangely altered. If the dog is actually mad, or it can not be decided with satisfactory certainty, it is almost a madness *not* to cauterize the part instantly with a white-hot iron, as this hurts less than if only red-hot, and is the only infallible remedy. Mad stones, whisky, ash-leaf infusions, drank most freely, have all often failed, proving that when their use was not followed by hydrophobia it was because the dog was only thought to be mad, or the system at the time was insusceptible to the poison, or the skin was unbroken. Madness begins in from two to twelve weeks, averaging from four to seven, rarely later than eight weeks.

